# Real-World Study of Cisplatin, Etoposide, and Bleomycin Chemotherapy Regimen in Gestational Trophoblastic Neoplasia

**DOI:** 10.1155/2021/6661698

**Published:** 2021-06-24

**Authors:** Ming Wang, Lianna Shen, Xiaohong Xu, Wei Duan, Jinwei Miao, Weimin Kong, Li Su, Yumei Wu

**Affiliations:** ^1^Department of Gynecologic Oncology, Beijing Obstetrics and Gynecology Hospital, Capital Medical University, Beijing, China; ^2^Department of Gynecologic and Obsterics, Shenzhen Sixth People's Hospital, Huazhong University of Science and Technology, Shenzhen, China

## Abstract

**Objective:**

Little observational data exist regarding the use of cisplatin, etoposide, and bleomycin (BEP) chemotherapy regimen in patients with gestational trophoblastic neoplasia (GTN).

**Methods:**

This is a retrospective study of 95 patients with GTN in our center from June/2010 to June/2018. All patients received at least 2 cycles of BEP chemotherapy. The primary outcomes were the rate of complete remission (CR) and overall survival (OS). The secondary outcomes were disease-free survival (DFS), pregnancy rates after BEP exposure, drug resistance rate, and other adverse events.

**Results:**

Of the 95 patients included, 66 (69.5%) patients received BEP as primary treatment and 29 (30.5%) were Salvage chemotherapy. The median age at diagnosis was 37 years (range 29.75-46) and 34 years (range 27-40) in two groups, respectively. The median WHO prognostic scores were 6 (range 3.5-8), and 77.32% of patients were FIGO stage III-IV in the primary treatment group. The median WHO prognostic scores were 5 (range 3-9), and 66.55% of patients were FIGO stage III-IV in the salvage treatment group. Median cycles of BEP treatment were 4 (3, 5) and 3 (2, 4) in two groups, respectively. In the primary chemotherapy group, 18.2% received additional hysterectomy, 4.5% received UAE for vaginal bleeding, and 1.52% received whole-brain radiotherapy. In the salvage chemotherapy group, 20.7% received hysterectomy, 6.9% received lobectomy, 3.4% received hysteroscopic lesion resection, and 3.4% received whole-brain radiotherapy. CR rates to initial chemotherapy were 86.4%, including 87.9% in the primary chemotherapy group and 82.8% in the salvage chemotherapy group. No predictive factor of chemotherapy resistance was identified. The rate of 5 year-DFS was 96.52% (95% CI 86.78–99.12) in the primary chemotherapy group and 92.44% (95% CI 73.02-98.06) in the salvage chemotherapy group. The rate of 5 year-OS was 98.31% (95% CI 88.57–99.76) and 95.65% (95% CI 79.93-99.38) in the two groups, respectively. During the treatment, neutropenia, thrombocytopenia, anemia, and liver dysfunction occurred in 80.3%, 6.1%, 25.8%, and 50% primary chemotherapy patients and 82.8%, 31%, 10.3%, and 86.2% salvage chemotherapy patients. In patients with fertility requirements, live birth rates were 100% (10/10) in primary chemotherapy patients and 80% (4/5) in salvage chemotherapy patients.

**Conclusions:**

BEP regimen was effective in the treatment of GTINs. The treatment was well tolerated, with no safety concerns on patients' fertility.

## 1. Introduction

The gestational trophoblastic disease is a spectrum of tumors that arise from placental tissue. Gestational trophoblastic neoplasia (GTN) refers to the invasive and malignant forms of gestational trophoblastic disease that include invasive mole, choriocarcinoma, placental-site trophoblastic tumor, and epithelioid trophoblastic tumor [1]. The incidence of GTN is ≤1/1000 deliveries, and the etiology of GTN is not well understood. The therapeutic decision is based on the International Federation of Gynecology and Obstetrics (FIGO) anatomic staging and prognostic scoring index [2]. Combination chemotherapy was recommended for primary treatment of high-risk (FIGO Stage III/IV or WHO score z7), salvage chemotherapy, resistant, or recurrent gestational trophoblastic neoplasms [3–5]. Combination chemotherapy with or without adjuvant radiotherapy or surgery could achieve a cure rate of 80%–90%.

EMA/CO is the most commonly used first-line therapy, with platinum-etoposide combinations, particularly EMA/EP (etoposide, methotrexate, dactinomycin/etoposide, and cisplatin). Alternatives, including TP/TE (paclitaxel, cisplatin/paclitaxel, and etoposide), BEP (bleomycin, etoposide, and cisplatin), FAEV (floxuridine, dactinomycin, etoposide, and vincristine), and FA (5-fluorouracil (5-FU) and dactinomycin), may be as effective as EMA/EP and associated with fewer side effects [3].

BEP regimen is recommended for treating high-risk, recurrent, or persisting GTN; however, high-quality evidence in support of this is still lacking. More studies about BEP chemotherapy in GTN are needed to enhance the body of therapeutic knowledge and capture the uncommon adverse events (AEs). Therefore, we conducted a prospective one-arm study of 95 patients to verify the safety and efficacy of the BEP regimen for GTN based on the patient-physician decision in a real-world setting.

## 2. Methods

### 2.1. Patient Selection

This is a retrospective single-arm study in which patients were recruited from a tertiary care hospital, the Beijing Obstetrics and Gynecology Hospital in China between July 2010 and July 2018. Subjects who were diagnosed with GTN and received BEP regimen chemotherapy were included in this study. The subjects were categorized as high risk according to the modified World Health Organization (WHO) scoring system, with extrauterine metastasis, or failed with other regimens. The trial was approved by the institutional ethics review committee (approval no: 2021-KY-052-01). The patients were stratified into two subgroups: primary chemotherapy group and salvage chemotherapy group based on having the previous chemotherapy or not.

### 2.2. Study Procedures

The electronic medical record system and hospital paper charts, pertinent data from the clinic, and inpatient services in the Department of Gynecological Oncology in Beijing Obstetrics and Gynecology Hospital were retrospectively reviewed.

Patients fulfilling the inclusion and exclusion criteria were included in the study. The chemotherapy protocol was chosen depending on the experience of the surgeons and patients' preference. Cisplatin 20 mg/m^2^ (30 mg) IV and etoposide 70 mg/m^2^ (100 mg) IV on days 1–5, as well as bleomycin 10 mg/m^2^ (15 mg) im on days 1-3 (see Supplementary Table 1). The regimens were repeated every 21-28 days. Pulmonary function testing was performed before initiation of therapy and every fourth dose thereafter.

To decrease the incidence of severe neutropenia, which occurs almost universally with these protocols, and avoid treatment delays, polyethylene glycol recombinant human granulocyte colony-stimulating factor (rhG-CSF) 3 mg/60Kg was administered in patients with previous III-IV neutropenia on days 3–14 of each treatment cycle, and a complete blood count was obtained at 3-4 days interval. No treatment course began unless the white blood cell count was ≥3,000/mL, and the platelet count was ≥100,000/mL. Anemia was treated with blood transfusion if the hemoglobin concentration was lower than 80 g/L. Serotonin 5-HT3 receptor antagonists were used to prevent nausea and vomiting. During chemotherapy, patients were monitored every 3 weeks for response and toxicity by physical examination, hematologic and chemistry profiles, and *β*-hCG levels.

### 2.3. Other Adjuvant Treatment

Surgery was considered to either excise sites of bulky and/or resistant tumors or less frequently to treat complications such as tumor hemorrhage or infection. Selective arterial embolization was used to control deep bleeding from the uterovaginal or other tumor sites. In cases with the isolated disease in the uterus or lungs, surgical resection was used in selected patients, for example, hysterectomy in patients who did not want to preserve fertility.

### 2.4. Radiotherapy

For patients with resistant or metastatic lesions in the brain, the chemotherapy regimen was adjusted and radiotherapy was given when necessary.

### 2.5. Follow-Up

During the therapies, history, and physical examination, complete blood counts, chemistry profiles, and *β*-hCG were done at the first day of each course of the treatment. Patients were considered to be responding by the decrease in *β*-hCG levels. After the *β*-hCG levels were normal, continue systemic therapy regimen for 2 cycles.

After the complete remission, follow-up *β*-hCG levels were measured monthly for 12 months, every 3 months during the second year and at 6-month intervals indefinitely thereafter. Patients were advised not to become pregnant for 1 year with the use of oral or barrier contraception to prevent pregnancy.

### 2.6. Outcome Measurements and Endpoints

The primary outcome was the rate of complete remission and overall survival through the end of June 2020. Complete remission was defined as three consecutive weekly B-hCG levels less than 10 mIU/mL. Besides, all tests showed (including physical examination and imaging studies) that the lesions had disappeared, clinical symptoms disappeared. Overall survival was defined as the time interval from the initiation of BEP chemotherapy to the date of death for any reason. Data of patients who were lost to follow-up were still included in the analysis.

The secondary outcomes were the rates of disease-free survival (DFS), pregnancy after BEP exposure, drug resistance, and other adverse events. Disease-free survival was defined as the length of time that the women remained disease-free. In this study, we will judge the disease-free survival time from the initiation of BEP chemotherapy. Drug resistance [5] was defined in patients whose serum *β*-hCG level was not decreased logarithmically or showed a platform-like level after two cycles of chemotherapy; metastases grow or does not shrink, or even the emerging new metastatic lesions. Recurrence was defined when the *β*-hCG level rose (except for pre-pregnancy) or other examination revealed new lesions. Adverse events evaluations were conducted by performing a CBC, urinalysis, renal and liver function tests, chest PA, and pulmonary function test (PFT). PFT was performed before initiation of therapy and in patients who complained of respiratory symptoms such as dyspnea and more than 4 cycles of BEP regimen. AEs were graded according to the Common Terminology Criteria for Adverse Events (version 5.0).

### 2.7. Statistical Analysis

Demographics, baseline characteristics, and safety results were summarized descriptively. Adverse events except fertility rates were evaluated using the full analysis set, defined as all patients included who received at least ≥1 cycle of BEP regimen, as well as the efficacy-evaluable analysis set, which included patients who received at least ≥1 cycle and who had at least 1 evaluable postbaseline tumor assessment. Fertility rates were evaluated using the fertility requirement analysis set, defined as patients included who did not receive hysterectomy and had the willingness to conceive.

Based on the previous study on BEP, we estimated 88.89% [6] patients achieved complete remission after the BEP chemotherapy, and the number of patients needed to verify the primary outcome was calculated to be 81 with a significance level of 0.05 and detection power of 0.80. Considering a 10% dropout rate in the retrospective study, a sample size of the patient included more than 89 was a reasonable estimation for the current study. For endpoint measurements, we performed the intention to treat (ITT) analysis, which was defined as analysis that included all included patients, including those with protocol deviations. Baseline characteristics and laboratory results were summarized for the three groups using descriptive statistics, including percentage, means ± standard deviation (SD), and 95% CI. For the quantitative variable, the *t*-test was used to compare group differences. For categorical variables, the chi-square test was used for group comparisons. The significance level was set at *P* < 0.05; all data were analyzed by SPSS 17.0 (SPSS, Inc., Chicago, IL).

## 3. Results

### 3.1. Study Population

**Figure 1:**
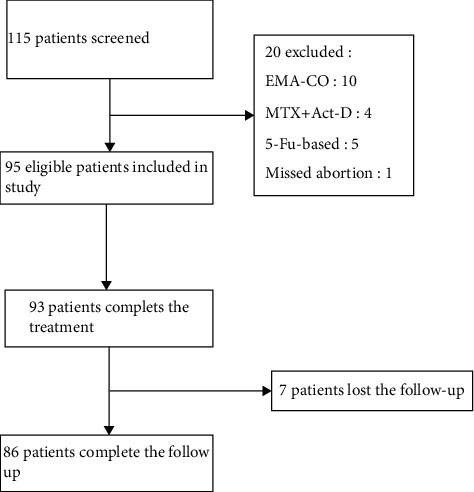
Patients' selections and study design.

Among the 115 patients reviewed, 95 patients were included in the study. 66 patients with gestational trophoblastic neoplasia and received BEP as the primary treatment and 29 patients who had failed primary treatment of other chemotherapy regimens for persistent or relapsed from remission selected BEP as the salvage therapy in the records of our center from June/2010 to June/2018 (Figure 1).

Patients ranged in age from 29.75 to 46 years (median = 37) in primary treatment patients and from 27 to 40 years (median = 34) in salvage chemotherapy patients. Human chorionic gonadotropin (hCG) levels before BEP treatment ranged from 4.08∗10^3^ to 1.28∗10^5^ mIU/mL (median = 1.53∗10^4^ mIU/mL) in primary treatment patients and from 417.65 to 6.50∗10^4^ mIU/mL (median = 3.87∗10^3^ mIU/mL) in salvage chemotherapy patients. 30.3% in the primary group and 24.1% in the salvage group had nonmolar antecedent pregnancies.

Metastatic sites were vagina 1.52% (1/66), cervix 3.03% (2/66), adnexa 4.55% (3/66), lung 75.8% (50/66), humerus 1.52% (1/66), in primary group and lung 62.1% (18/29), and brain 3.45% (1/29) in salvage group. 15.2% (10/66) in the primary treatment group and 34.5% (10/29) patients in the salvage group with the refractory disease in the uterus had no evidence of metastasis. FIGO stage in the primary treatment group was as follows: 15.2% had stage I disease, 7.6% had stage II, 75.8% had stage III, and 1.52% had stage IV disease. FIGO stages in the salvage treatment group were as follows: 34.5% had stage I disease, 0% had stage II, 62.1% had stage III, and 3.45% had stage IV disease. World Health Organization (WHO) prognostic scores ranged from 3.5 to 8 (median = 6) in patients who received BEP as primary treatment, and 3 to 9 (median = 5) in patients who received BEP as salvage treatment (Table 1).

#### 3.1.1. Response to BEP Chemotherapy

The median (IQR) duration of chemotherapy cycles was 4 (3, 5) in patients who received BEP as primary chemotherapy. Of 66 primary chemotherapy patients, 18.2% underwent additional hysterectomy, 4.5% underwent UAE for vaginal bleeding, and 1.52% underwent whole-brain radiotherapy. 87.9% (58/66) had a complete response and 98.5% (65/66) survived. 25.8% (17/66) patients received another regimen during the treatment including 12.1% (8/66) patients who did not have CR, 4.5% (3/66) who did not tolerate the adverse events of BEP, and 9.1% (6/66) for the absence of bleomycin. 87.5% (7/8) patients who failed primary treatment with BEP had a complete clinical response to secondary chemotherapy with EMA-CO, EMA-EP, or 5-FU + Act-D (Table 2).

29 patients with persistent/relapsed gestational trophoblastic neoplasia received BEP salvage chemotherapy. The BEP regimen yielded complete responses in 82.8% (24/29) patients, including 34.5% (10/29) patients who had failed 5-Fu based combined regimen, 27.6% (8/29) patients who had failed methotrexate/actinomycin D/5-Fu single-drug regimen, and 37.9% (11/29) patients who had failed other combined regimens. Of 29 salvage chemotherapy patients, 20.7% received additional hysterectomies, 6.9% underwent the resection of resistant pulmonary nodules, 3.4% received transcervical resection of focus, and 3.4% underwent whole-brain radiotherapy. 27.6% (8/29) patients received other regimens during the treatment including 17.2% (5/29) patients who did not have CR and 10.3% (3/29) who did not tolerate the adverse events of BEP. 80% (4/5) patients who failed primary treatment with BEP had a complete clinical response to other regimens (Table 2).

No predictive factor of chemotherapy resistance was identified in two subgroups.

#### 3.1.2. Overall and Disease-Free Survival

**Figure 2:**
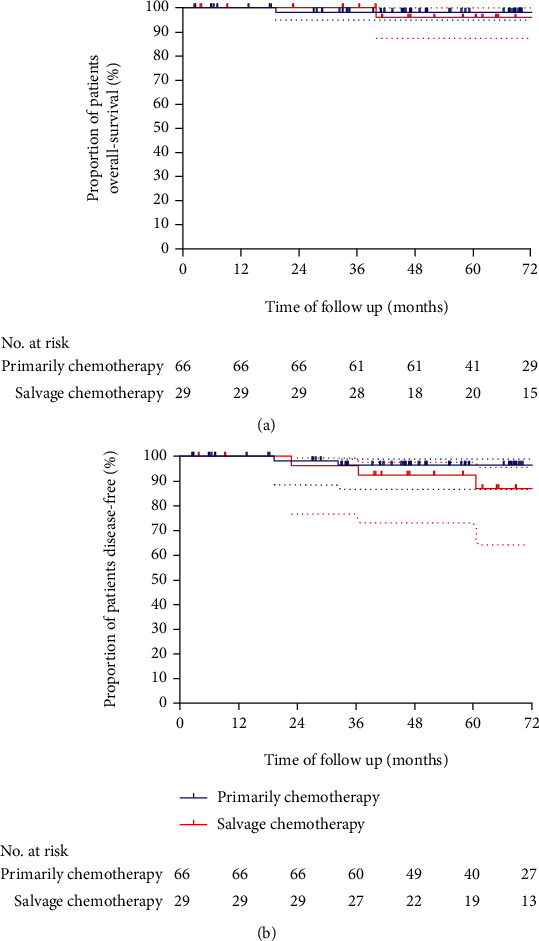
Kaplan–Meier estimates of overall survival and disease-free survival. (a) shows the Kaplan–Meier plot for overall survival, measured from the date of initiation of chemotherapy to the date of death or the date that the patient was last known to be alive. (b) shows the cumulative incidence curves for disease-free survival, measured from the date of surgery to the date of death or the date that the patient was last known without recurrence. A Cox proportional-hazards model was used to determine the hazard ratio and 95% confidence interval. Tick marks indicate censored data.

The median duration of follow-up was 68.70 months (range, 47.23-80.96 months) in the primary treatment group and 68.86 months (range, 49.55-91.97months) in the salvage chemotherapy group. During the follow-up, 7 patients were lost to follow-up. 2 patients died of the disease. In all treated patients, the overall survival rate at 5 years was 98.31% (95% CI 88.57–99.76) in the primary chemotherapy group and 95.65% (95% CI 79.93-99.38) in the salvage chemotherapy group, respectively (Figure 2(a)). Only 1 patient died of multiple metastasis 19 months in the primary chemotherapy group after the initial treatment. The remaining patients were placed into lasting remission (Table 3). 1 patient in the salvage chemotherapy group died 40 months after the BEP treatment. The rate of disease-free survival at 5 years was 96.52% (95% CI 86.78–99.12) in the primary chemotherapy group and 92.44% (95% CI 73.02-98.06) in the salvage chemotherapy group (Figure 2(b)). 5 patients had a recurrence, including 2 in the primary chemotherapy group and 3 in the salvage chemotherapy group. Patients who had a recurrence in the primary chemotherapy group were 1 simple *β*-HCG elevating and 1 multiple metastasis. Patients having a recurrence in the salvage chemotherapy group were 1 in lung, 1 in brain, and 1 in pelvic cavity. The patients with recurrence in lung were further returned to the general hospital with thoracic surgery. As expected, the patients achieved complete remission after the residual disease resected. 1 patient developed brain and lung metastasis 5 years after 6 courses of salvage BEP, and she received brain radiation, lobectomy, and other regimen chemotherapy. She is now in persisting remission but has epilepsy as the sequelae of brain metastasis and treated with Keppra. The patients with pelvic relapsed achieved complete remission after chemotherapy of EMA/CO regimen. She is now in the additional chemotherapy of EMA/CO.

### 3.2. BEP Chemotherapy Side Effects

There were 10 itemized AEs recorded based on the patients' complaints, physical findings, and laboratory abnormalities, which included neutropenia, thrombocytopenia, anemia, liver dysfunction, renal toxicity, epilepsy, leukemia, femur head necrosis, pulmonary toxicity, and POF (as shown in Table 2). The study did not show a severe allergic reaction and obvious heart, liver, lung, and kidney dysfunction in patients. In patients with fertility requirements, live birth rates were 100% (10/10) in primary chemotherapy patients and 80% (4/5) in salvage chemotherapy patients. 1 patient underwent recurrent IVF failure for POI after the chemotherapy.

The most common treatment-related adverse events of were bone marrow suppression, including 80.3% (53/66) neutropenia, 50% (33/66) ALT elevation, 25.8% (17/66) anemia, 6.1% (4/66) thrombocytopenia, 1.5% (1/66) leukemia, 1.5% (1/66) in primary chemotherapy patients, 86.2% (25/29) ALT elevation, 82.8% (24/29) neutropenia, 31% (9/29) thrombocytopenia, 10.3% (3/29) anemia, and 3.4% (1/29) epilepsy in salvage chemotherapy patients, respectively. No treatment-related patient deaths or cases of BEP or acute allergic reaction occurred. However, most AEs on bone marrow suppression reported were grade I-II and were observed in this study, including 55.8% (43/77) neutropenia, 70% (14/20) anemia, and 84.6% (11/13) thrombocytopenia. Except for 1 case in which severe bone marrow suppression occurred leading to the cessation of chemotherapy, the other patients were not influenced by the side effects in the course of chemotherapy. The 57 cases with ALT elevation observed during chemotherapy were 59.2 (IQR: 48.1-84.9) IU/L (grade I-II AE). 1 patient who received 6 courses of BEP regimen and 2 additional courses of EMA-CO for drug resistance had leukemia 11 months after primary BEP chemotherapy and received bone transplantation. She is now being well (Table 3).

## 4. Discussion

In this study, we report data on BEP therapy on the treatment of GTN in high-risk, relapsed, or uncontrolled patients in a real-world setting. To our knowledge, this is the largest real-life study with 96 subjects included to evaluate the effects and safety of BEP chemotherapy in patients with GTN. Our results indicated that the BEP regimen was effective and well-tolerated concerns on patients' fertility. In combination with additional treatment, the use of the BEP regimen yielded a 97.7% successful rate of treating MTCT in the on-protocol analysis in our real-life setting. Currently, the treatment principle of high-risk GTN is combinational chemotherapy as the first choice, and on this basis, radiotherapy and (or) other treatments such as surgery are given when appropriate [1]. EMA/CO is currently the most widely used first-line combination chemotherapy for high-risk GTN, although this regimen has not been rigorously compared to other combinations such as EMA/EP, BEP, TP/TE, or 5-FU based regimen.

In previous studies, EMA/EP salvage treatment following EMA/CO treatment failure, cure rates ranged from 66.6% to 84.9% [7–9]. However, EMA/EP was associated with significant myelosuppression and hepatotoxicity, leading to treatment delays and dose reductions. The taxane-containing regimen, TP/TE, was found to be associated with comparable cure rates to EMA/EP (70% of 10 patients) but with relatively reduced toxicity and no dose delays or reductions [10]. In a study of 222 patients, FA (5-FU and dactinomycin) was found to be effective as primary treatment for low- and high-risk GTN (with remission rates of 99% and 84%, respectively), except in patients with extensive metastases [11]. BEP regimen is recognized as the preferred choice in the treatment of malignant ovarian germ cell tumors (including primary ovarian choriocarcinoma). The regimen has been reported in the literature for its good effect in early, advanced, and recurrent malignant ovarian germ cell tumors. Song et al. examined the safety and efficacy outcomes on high-risk gestational trophoblastic tumors who were treated with BEP (*n* = 45 for endpoint analysis). The total complete remission rate of the BEP regimen was 88.89% (40/45). Five patients developed drug resistance after an average of 4.8 courses of BEP, and the regimen converted EMA/CO. Ultimately, four cases achieved CR and one case died of cancer. One of 45 patients who developed grade IV bone marrow suppression and worsened pulmonary fibrosis after chemotherapy. None of the survival patients developed a secondary tumor during the follow-up [6]. BEP is favored as salvage therapy in FA-resistant GTN, citing greater convenience compared with EMA/CO, and reporting a remission rate of 80% (10 out of 12) in high-risk cases. We observed 87.9% CR in primary chemotherapy GTNs and 82.8% in salvage chemotherapy GTNs. One patient died early of massive metastasis half a year after diagnosis, 1 died of disease relapse 40 months after treatment initiation, and 86 were alive until now except 7 patients who were lost to follow-up. Our data, together with the previous study, further support the use of BEP chemotherapy had a similar response to other combination regimens.

The adverse reactions of BEP are relatively mild, and the cure rate of malignant ovarian germ cell tumors could reach over 90%. Following these strategies, most patients will be cured, and the vast majority will still be able to give birth [12, 13]. In terms of safety data, salvage BEP-treated patients experienced a less cycle but higher adverse events (besides anemia) than primary BEP-treated patients, such as neutropenia, thrombocytopenia, and liver injury. The myelosuppression may be related to previous other regimens and BEP chemotherapy together. These observations yielded similar safety profiles with those data published in the aforementioned studies. However, our study showed 1 leukemia and 1 femur head necrosis after BEP chemotherapy. After bone marrow transplantation, the patients with leukemia were CR until now. No evidence of decreased fertility after chemotherapy for trophoblastic neoplasia was observed. Only one patient underwent POI after chemotherapy and failed for several IVF-ET. No congenital abnormalities were observed in our study.

In the traditional BEP regimen, bleomycin was used weekly for 12 weeks [14]. In our center, the dosage and interval of bleomycin were changed for the concerns of developing pulmonary fibrosis. The bleomycin was used in three weeks intervals, and the dosage was 45 mg per cycle in our study. Then, the accumulating dosage of bleomycin of 4 cycle chemotherapy was 180 mg not 360 mg in the traditional regimen, which was lower than the restriction dosage of pulmonary fibrosis. And, no pulmonary fibrosis was found.

However, no control group was obtained in our study for patient characteristics in our study varying widely about the type and number of previous regimens administered, risk scores, and other factors. Then, direct comparisons of BEP with other regimens in a larger sample are needed for patients with GTN.

In conclusion, our results indicated that the BEP regimen was effective in the treatment of GTINs. The treatment was well tolerated, with no safety concerns on patients' fertility.

## Figures and Tables

**Table 1:** Baseline Values of GTN patients who received BEP chemotherapy (*n* = 95).

*N* (percentile) Mothers	Primary treatment group (*n* = 66)	Salvage treatment group (*n* = 29)
Median age (range)	37 (29.75, 46)	34 (27, 40)
<40 years	36 (54.5)	21 (72.4)
≥40 years	30 (45.5)	8 (27.6)
Antecedent pregnancy		
Hydatidiform mole	46 (69.7)	22 (75.9)
Abortion	14 (21.2)	3 (10.3)
Term pregnancy	6 (9.1)	4 (13.8)
Interval		
<4 months	43 (65.2)	18 (62.1)
4–6 months	10 (15.2)	6 (20.7)
7–12 months	2 (3.0)	2 (6.9)
>12 months	11 (16.7)	3 (10.3)
Pretreatment *β*-hCG level		
<1,000	5 (7.6)	10 (34.5)
1,000–<10,000	22 (33.3)	5 (17.2)
10,000–<100,000	21 (31.8)	10 (34.5)
>100,000	18 (27.3)	4 (13.8)
Previous chemotherapy		
Yes	0	29
No	66	0
Tumor size (in cm)		
<3	34 (51.5)	21 (72.4)
3-5	18 (27.2)	4 (13.8)
≥5	14 (21.2)	4 (13.8)
Site of metastasis		
Vagina	1 (1.52)	0 (0)
Cervix	2 (3.03)	0 (0)
Adnexa	3 (4.55)	0 (0)
Lung	50 (75.8)	18 (62.1)
Brain	0 (0)	1 (3.45)
Humerus	1 (1.52)	0 (0)
FIGO stage		
I	10 (15.2)	10 (34.5)
II	5 (7.6)	0
III	50 (75.8)	18 (62.1)
IV	1 (1.52)	1 (3.45)
Prognostic score	6 (3.5,8)	5 (3,9)
Low-risk	35 (53)	18 (62.1)
High-risk	31 (47)	11 (37.9)

∗Note: BEP: cisplatin, etoposide, and bleomycin; GTN: gestational trophoblastic neoplasia; FIGO: International Federation of Gynecology and Obstetrics.

**Table 2:** Efficacy of BEP chemotherapy in GTN patients (*n* = 95).

*N* (percentile)	Primary treatment group (*n* = 66)	Salvage treatment group (*n* = 29)
Course of BEP	4 (3, 5)	3 (2, 4)
Additional treatment	15 (22.7)	10 (34.5)
Hysterectomy	12 (18.2)	6 (20.7)
UAE	3 (4.5)	0 (0)
Radiation	1 (1.52)	1 (3.4)
Lobectomy	0 (0)	2 (6.9)
Hysteroscopic lesion resection	0 (0)	1 (3.4)
Response		
CR	58 (87.9)	24 (82.8)
PR	8 (12.1)	5 (17.2)
Reasons to change BEP regimen	17 (25.8)	8 (27.6)
Resistance	8 (12.1)	5 (17.2)
Nontolerable	3 (4.5)	3 (10.3)
Strengthen for no bleomycin or restriction	6 (9.1)	0 (0)

∗Note: BEP: cisplatin, etoposide, and bleomycin; GTN: gestational trophoblastic neoplasia; UAE: uterine artery embolism; CR: complete remission; PR: partial remission.

**Table 3:** Adverse events reported in the study (*n* = 95).

Adverse events *N* (%)	Primary treatment group (*n* = 66)	Salvage treatment group (*n* = 29)
Neutropenia	53 (80.3)	24 (82.8)
Grade 1/2	31 (50)	12 (41.4)
Grade 3/4	22 (33.3)	12 (41.4)
Thrombocytopenia	4 (6.1)	9 (31)
Grade 1/2	4 (6.1)	7 (24.1)
Grade 3/4	0 (0)	2 (6.9)
Anemia	17 (25.8)	3 (10.3)
Grade 1/2	13 (19.7)	1 (3.4)
Grade 3/4	4 (6.1)	2 (6.9)
Liver dysfunction	33 (50)	25 (86.2)
Grade I/II (1-2.5, 2.5-5)	33 (50)	25 (86.2)
Grade III/IV (5-20, >20)	0 (0)	0 (0)
Renal toxicity	0 (0)	0 (0)
Epilepsy	0 (0)	1 (3.4)
Leukemia	1 (1.5)	0 (0)
Femur head necrosis	1 (1.5)	0 (0)
Pulmonary toxicity	0 (0)	0 (0)
POF	0 (0)	2 (6.90)
Pregnancy rates	10/10 (100)	4/5 (80)

∗Note: AEs: adverse events; POF: premature ovarian failure.

## Data Availability

The datasets used and/or analyzed during the current study are available from the corresponding author on reasonable request.

## Abbreviations

BEP: Cisplatin, etoposide, and bleomycin
GTN: Gestational trophoblastic neoplasia
CR: Complete remission
FIGO: International Federation of Gynecology and Obstetrics
AEs: Adverse events
OS: Overall survival
DFS: Disease-free survival
ITT: Intention to treat analysis.

## Supplementary Materials

Supplementary Materials Supplementary Table 1 showed the BEP treatment regimen applied in this study.
