# Supplementary material for: Real-World Study of Cisplatin, Etoposide, and Bleomycin Chemotherapy Regimen in Gestational Trophoblastic Neoplasia
Source: Biomed Res Int. 2021 Jun 24;2021:6661698. doi: 10.1155/2021/6661698 (PMC8249144; doi:10.1155/2021/6661698)
Supplement: Supplementary Materials — Supplementary Table 1 showed the BEP treatment regimen applied in this study. [file 6661698.f1.docx]

**Suppl table 1. The BEP treatment regimen applied in this study**

|  | **Dosage(mg)** | **Intervals** (per 3 weeks) |
| --- | --- | --- |
| **Cisplatin** | 30mg (20 mg/m2) | Day 1-5 |
| **Etoposide** | 100mg (70-100 mg/m2 ) | Day 1-5 |
| **Bleomycin** | 15mg(10mg/m2) | Day 1-3 |
